# Neuropeptide reporter assay for serum, capillary blood and blood cards

**DOI:** 10.1016/j.mex.2020.100985

**Published:** 2020-07-04

**Authors:** U. Schreiber, C. Engl, M. Bayer, S. König

**Affiliations:** IZKF Core Unit Proteomics, University of Münster, Germany

**Keywords:** Bradykinin, Substance P, Thin-layer chromatography, Dried blood spots, Protease activity

## Abstract

In the associated main paper (JPBA 2019), substance P was shown to be a valuable neuropeptide reporter substance to monitor the protease activity of serum. The assay was developed based on the predecessor assay using bradykinin (JPBA 2017). Both neuropeptides are of interest in inflammation and pain research and were thus explored for use with capillary blood and blood cards. Here, we present the protocols and set them in perspective to above neuropeptide assays for serum.

• Neuropeptide reporter substance protease activity assay for use with fresh and dried blood.
• Dabsylated Substance P and bradykinin are substrates of angiotensin-converting enzyme and other proteases.
• Neuropeptides of interest in inflammation and pain.

## Graphical abstract

**Figure:**
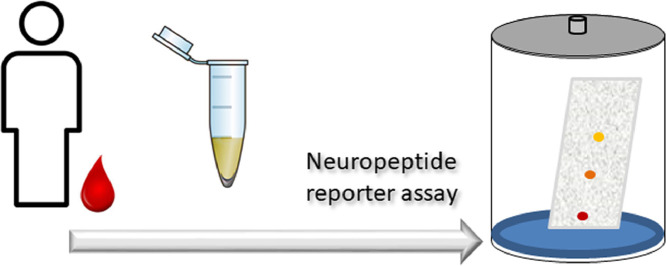

Specifications Table

**Table:** 

Subject Area	Biochemistry, Genetics and Molecular Biology
More specific subject area	Enzyme activity
Method name	Neuropeptide reporter substance protease activity assay (NRS-PAA)
Name and reference of original method	Dabsyl-bradykinin protease activity assay (DBK-PAA)
	M. Bayer, S. König, A vote for robustness: Monitoring serum enzyme activity by thin-layer chromatography of dabsylated bradykinin products. J. Pharmaceut. Biomed. Anal. 143 (2017) 199–203.
Resource availability	Dabsylated peptides were ordered from Peptide Specialty Laboratories (Heidelberg, Germany)
	*Microcapillaries (Drummond Microcaps) allowed replicate collection with a defined volume (*3 or 5 µl*)*
	*Blood cards (Whatman 903 Protein Saver Card)*

This MethodX article supplements work were we show the use of dabsylated substance P (DSP) as a neuropeptide reporter substance (NRS) for the monitoring of protease activity (PA) in serum [1]. This article is based on the assay developed for the use of dabsylated bradykinin (DBK) for the same purpose [2], which was already employed in a clinical study [3]. Both peptides are of importance in research on inflammation and pain [4]. We have, therefore, evaluated the application of the assay with respect to other types of sampling such as capillary blood and blood cards in order to extend the application range of the assay, which was only used with serum so far [1, 2]. Sampling thus becomes much easier, because little blood is needed, and outpatient studies are feasible as no trained personnel is necessary to prepare the blood cards. Moreover, collecting the drop of blood from regions of interest such as a swollen limb is a true option and may reduce the need for skin biopsies.

The aim of this article is to describe the workflow for the use of a drop of blood, either used fresh or dried on blood cards, and set it in relation to the methods for serum for improved understanding. Validation data are presented in the associated Data in Brief article [5].

The NRS-PA assay (NRS-PAA) is based on four general steps (exemplified for DBK-PAA with capillary blood in Fig. 1):

(1) Incubation of dabsylated neuropeptide with body fluid or other biochemical preparation where PA is of interest;
(2) Purification of enzymatic fragments;
(3) Thin-layer chromatography (TLC) for separation of enzymatic fragments;
(4) Scanning and data analysis.

**Fig. 1:**
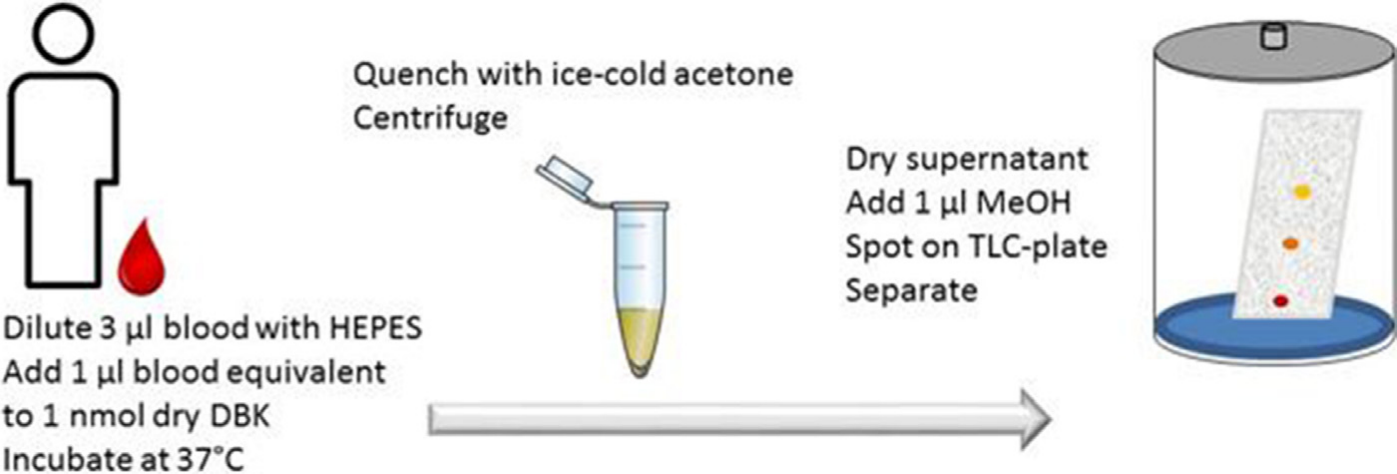
Workflow of the DBK-assay using capillary blood.

An early extensive protocol for DBK-PAA is available for the interested user [6]. It was however modified and simplified later [2]. DSP-PAA [1] differs slightly from DBK-PAA and more adaptations were required for fresh and dried capillary blood. Following initial in-house synthesis of DBK and DSP [1, 2] we now order the labeled peptides from Peptide Specialty Laboratories (Heidelberg, Germany). Also, different analysis software was tested [2, 6]; currently we prefer JustTLC (Sweday, Sodra Sandby, Sweden, Fig. 2) [1]. Table 1 shows the current state-of-the-art and compares all methods.

**Fig. 2:**
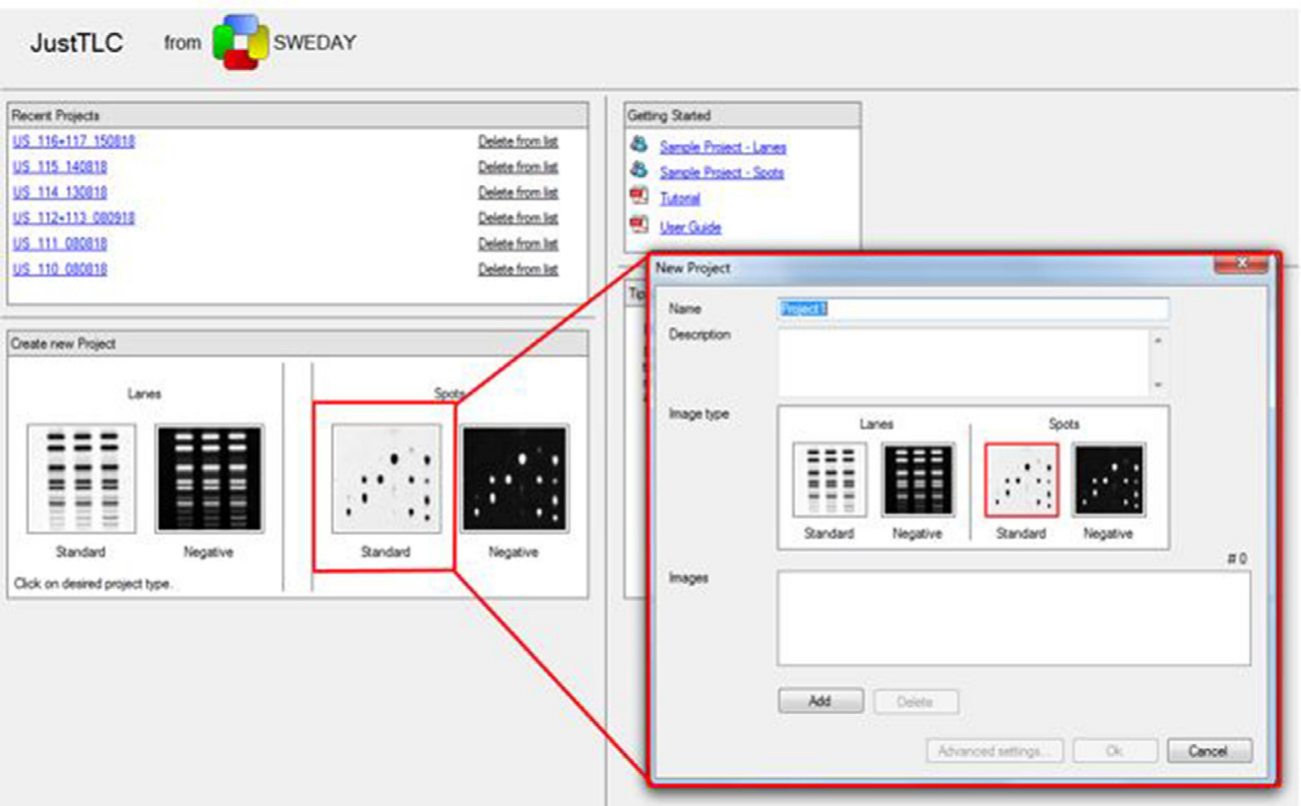
TLC data analysis using the software JustTLC.

**Table 1:** Comparison of all NRS-PAA approaches using the latest protocol.

	DBK/serum [2]	DBK/capillary blood	DBK/dried blood spots	K3DSP/ serum [1]	K3DSP/dried blood spots

NRS, dry/nmol	0.5	1	1	1 in 5 µl water	1
Extraction		10 µl HEPES-buffer/ 1 µl blood	10 µl HEPES-buffer/ 1 µl blood		10 µl HEPES-buffer/1 µl blood
Sample/µl	3	1 µl equivalent (10 µl HEPES solution)	1 µl equivalent (10 µl HEPES solution)	7.5	1 µl equivalent (10 µl HEPES solution)
Incubation temperature/°C	37	37	37	40	37
Typical incubation time/h	1	1	1	2	1
Precipitation at −20 °C, with x µl acetone for x h	18 µl, 2 h	60 µl, 1 h	60 µl, 1 h	78 µl, 1 h	60 µl, 1 h
Dissolve dry supernatant in 1 µl	methanol	ethanol (methanol, Fig. 2)	ethanol (methanol, Fig. 2)	methanol	ethanol
TLC mobile phase/CHCl_3_/methanol/ H_2_O/CH3COOH, v/v/v/v	11: 4: 0.6: 0.09	11: 4: 0.6: 0.09	11: 4: 0.6: 0.09	11: 3.5: 0.6: 0.18	11: 4: 0.6: 0.09

Blood was obtained from healthy volunteers observing the declaration of Helsinki. For the sampling of capillary blood, Microcapillaries (Drummond Microcaps) allowed replicate collection with a defined volume (1, 3 or 5 µl) and were emptied into vials or onto blood cards (Whatman 903 Protein Saver Card, Fig. 3). Blood cards were air-dried for 1–2 h and then frozen until further use. Individual blood spots were extracted with 10 times the volume of HEPES-buffer (50 mM HEPES, 300 mM NaCl, pH 7.4) for 15 min at room temperature. For the investigation of fresh blood, nine times the volume of HEPES-buffer was added. In both cases, the solution equivalent of 1 µl blood was subsequently incubated with 1 nmol dabsylated bradykinin (DBK; 37 °C, 60 min). For further sample preparation, six times the volume of ice-cold aceton was added followed by storage at −20 °C for 1 h. The supernatant was removed and dried for separation by TLC as described [2]. The dry residue was dissolved in 1 µL ethanol and spotted onto a TLC-plate; the sample vial was rinsed with another 1 µl which was added to the same spot.

**Fig. 3:**
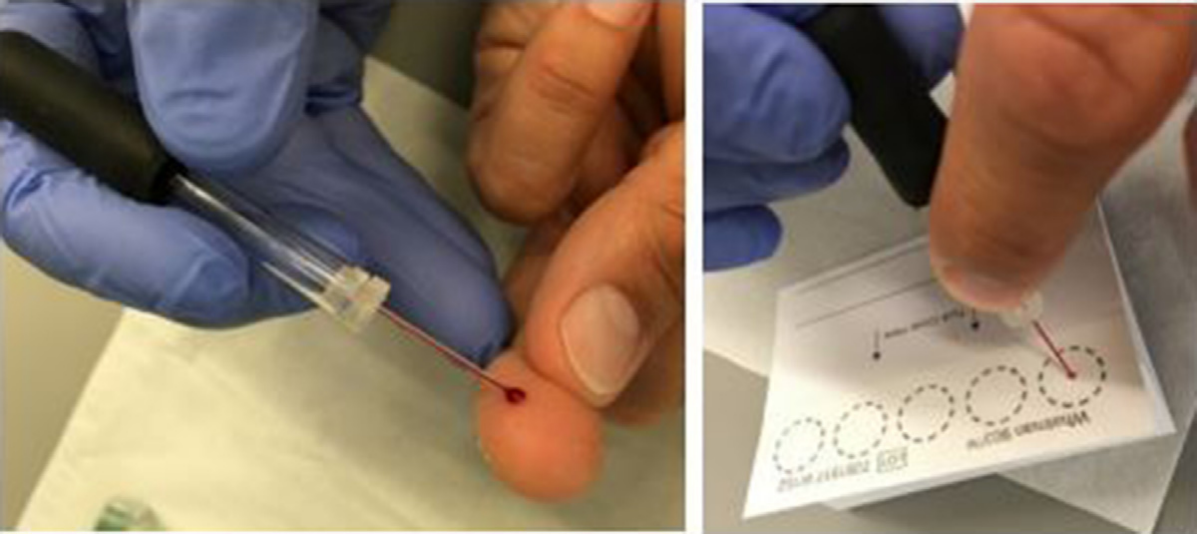
Collection of capillary blood and spotting onto blood cards.

For healthy probands, 70–90% of DBK1–9 were degraded in 60 min [5]. For DBK, the observed fragment signals were the same as before (DBK1–5, DBK 1–8) [2] albeit at higher intensity for lower sample volume. An exception was the observation of fragment DBK1–3 in fresh blood (Fig. 4) when diluted with water instead of buffer, which is of interest biochemically as this fragment was not described so far. In 60 min-experiments, the standard deviation for 12 × 3 technical replicates (DBK1–9: mean relative intensity 0.17, StD 0.05, DBK1–8: 0.16, StD 0.05, DBK1–5: 0.68, StD 0.09) was much lower than that for biological replicates enabling its use in the first place. Variation increased, when the dried blood spots were stored at room temperature for more than three days. Then, also the protease activity of the sample decreased. Conclusively, longer storage at room temperature should be avoided, but mailing of the samples is still possible - extended transfer times can be achieved with cooling.

**Fig. 4:**
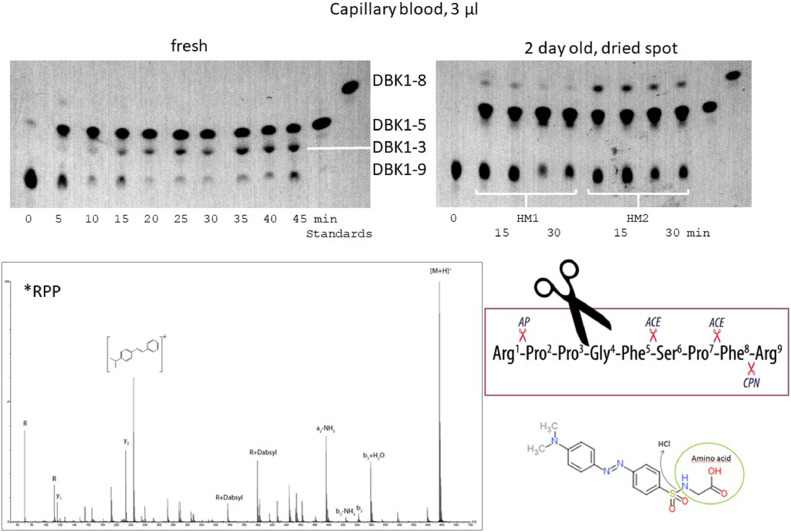
DBK-PAA for fresh and dried capillary blood (here: 3 µl, dissolvation in methanol for TLC) shown with reference substances. The formation of DBK1–3 was verified using mass spectrometry. HM – healthy male.

## Declaration of Competing Interest

The authors declare that they have no known competing financial interests or personal relationships that could have appeared to influence the work reported in this paper.
